# Chronic Arsenic Poisoning Probably Caused by Arsenic-Based Pesticides: Findings from an Investigation Study of a Household

**DOI:** 10.3390/ijerph13010133

**Published:** 2016-01-16

**Authors:** Yongfang Li, Feng Ye, Anwei Wang, Da Wang, Boyi Yang, Quanmei Zheng, Guifan Sun, Xinghua Gao

**Affiliations:** 1 Research Center of Environment and Non-Communicable Disease, School of Public Health, China Medical University, No. 77 Puhe Road, Shenyang North New Area, Shenyang, Liaoning 110122, China; liyongfang_17@163.com (Y.L.); wangda@mail.cmu.edu.cn (D.W.); boyycmu@163.com (B.Y.); qmzheng@mail.cmu.edu.cn (Q.Z.); 2 Yunnan Institute of Endemic Disease Control and Prevention, No. 5 Wen Hua Road, Dali, Yunan 671000, China; feng_ye9@163.com (F.Y.); waw1978@163.com (A.W.); 3 Department of Dermatology, No. 1 Hospital of China Medical University, Shenyang, Liaoning 110001, China

**Keywords:** arsenic, arsenic-based pesticides, skin lesions, chronic arsenic poisoning

## Abstract

In addition to naturally occurring arsenic, man-made arsenic-based compounds are other sources of arsenic exposure. In 2013, our group identified 12 suspected arsenicosis patients in a household (32 living members). Of them, eight members were diagnosed with skin cancer. Interestingly, all of these patients had lived in the household prior to 1989. An investigation revealed that approximately 2 tons of arsenic-based pesticides had been previously placed near a well that had supplied drinking water to the family from 1973 to 1989. The current arsenic level in the well water was 620 μg/L. No other high arsenic wells were found near the family’s residence. Based on these findings, it is possible to infer that the skin lesions exhibited by these family members were caused by long-term exposure to well water contaminated with arsenic-based pesticides. Additionally, biochemical analysis showed that the individuals exposed to arsenic had higher levels of aspartate aminotransferase and γ-glutamyl transpeptidase than those who were not exposed. These findings might indicate the presence of liver dysfunction in the arsenic-exposed individuals. This report elucidates the effects of arsenical compounds on the occurrence of high levels of arsenic in the environment and emphasizes the severe human health impact of arsenic exposure.

## 1. Introduction

Arsenic, a toxic element, is widely distributed in the environment. Accumulated evidence shows that long-term exposure to arsenic is associated with cancers of the skin, lung, bladder, liver and kidney. The International Agency for Research on Cancer has classified arsenic as a group I human carcinogen [1]. In addition to cancers, long-term exposure to arsenic is also associated with various non-cancer adverse effects, including skin lesions [2], cardiovascular diseases [3,4] diabetes [5], birth defects [6], abortion [7] and cognitive impairment [8]. Despite these notorious toxic effects of arsenic in humans, it is still widely used in both agriculture and industry [9,10,11,12]. The expanded use of arsenic has increased the possibility of toxic exposure, posing a corresponding new health risk to humans. Arsenic-based pesticides are the best examples of agricultural applications of arsenic. During the beginning and middle of the 20th century, inorganic arsenic-based pesticides, including lead arsenate, copper arsenate and calcium arsenate, were extensively used to control insects. It has been reported that approximately 15 million pounds of arsenic-based pesticides, which is equivalent to 6.8 million kilograms of arsenic, were applied to New Jersey (USA) soils between 1980 and 1990 [13]. This large amount of inorganic arsenic-based pesticides has led to serious arsenic contamination in the environment [14,15]. A number of acute and sub-acute arsenic intoxication cases have been reported [16,17]. Feinglass *et al.* described a case in which 13 individuals from Perham (MN, USA) were exposed to well water containing from 11,800 to 21,000 μg/L of arsenic for nearly two months, resulting in sub-acute arsenic toxicity in 11 individuals [16]. This severe contamination occurred because the well was inadvertently drilled in an area that had been previously used to prepare and store arsenic-laced grasshopper bait. Another outbreak of fatal arsenic poisoning, which was reported by Armstrong *et al.*, occurred among nine members of a family, of whom eight developed gastrointestinal symptoms, four developed encephalopathy, and two died [17]. The well water used by the family contained 108,000 μg/L of arsenic, most likely derived from waste arsenical pesticides that had been previously placed in the vicinity of the well. In contrast, here, we report a chronic arsenic poisoning event that occurred in a household. The event was probably caused by the family members’ chronic exposure to well water contaminated with arsenic-based pesticides. Approximately one-half of the household members were diagnosed with arsenic-related skin manifestations, including skin cancer. We hope that this report will raise awareness of the important impacts of arsenic-based compounds on the occurrence of high arsenic levels in the environment and also on human health.

## 2. Materials and Methods

### 2.1. Background

In 2013, our research group, together with the Institute of Endemic Disease Control and Prevention in Yunnan Province, performed screening of arsenic-contaminated areas in Midu County, China. A 43-year-old woman was diagnosed with obvious skin manifestations that appeared to be symptoms of chronic arsenic poisoning.

**Figure 1:**
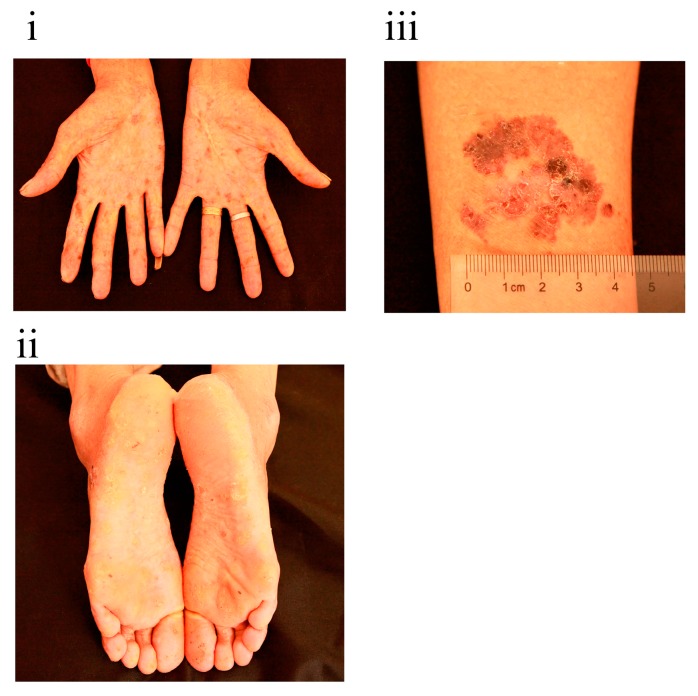
Clinical appearance of the woman we first identified: (**i**) palms; (**ii**) soles; (**iii**) right arm.

As shown in Figure 1, significant pigmentation, de-pigmentation and hyperkeratosis were found on her palms and soles. A 3-cm scaly, erythematous plaque was also detected on her right arm. The woman told us that her family members had similar skin features, some of which were more serious. We then immediately went to the woman’s family to initiate an investigation.

### 2.2. Investigation

All of the woman’s family members, except for those working outside of the village, were examined for symptoms of chronic arsenic poisoning, including pigmentation and de-pigmentation on the trunk and hyperkeratosis on the palms of the hands and soles of the feet. Skin biopsies were performed on nine family members to confirm skin malignancy. Detailed interviews with the family members were also performed to obtain information regarding age, sex, and dates of birth and marriage. Samples of water previously and currently used by the family, as well as some groundwater samples near the family’s residence, were collected for arsenic analysis. Additionally, 25 family members agreed to provide their first void urine in the morning and fasting blood samples for further analysis. 

#### 2.2.1. Urinary Arsenic Speciation Measurement

Each family member’s first void urine was collected in the morning in a 15-mL polypropylene tube and kept in a cooler. All samples were stored on dry ice and transferred by air to the Laboratory of Arsenic Analysis at China Medical University (Shenyang, China). The urine samples were stored at −80 °C and were then measured for urinary arsenic speciation. The analytical method applied in our study has been frequently used for detection urinary arsenic speciation in our laboratory, and the detailed procedures have been described in our previous reports [18,19,20]. Briefly, a 1-mL urine sample was thawed from −80 °C to room temperature. Then, a 2 normality (N) NaOH solution was added to the urine sample for digestion at 100 °C for 3 h, followed by dilution with Milli-Q water. The samples were stirred once per hour. An atomic absorption spectrophotometer (AA-6800, Shimadzu Co. Kyoto, Japan) equipped with an arsenic speciation pretreatment system (ASA-2sp, Shimadzu Co.) was used to detect inorganic arsenic (iAs), monomethylarsonic acid (MMA) and dimethylarsinic acid (DMA). Aliquoted samples were used for each assay. Arsenic speciation was determined by the well-established hydride generation of volatile arsines, followed by cryogenic separation in liquid nitrogen. The absorbance of arsenic in the treated urine samples was determined at a wavelength of 193.7 nm. The limit of detection for the three arsenic species (iAs, MMA, and DMA) was 1 ng, and the coefficient of variation was <5%. Quality control for arsenic determination included analysis of Standard Reference Material of freeze-dried urine (SRM 2670, National Institute of Standards and Technology (NIST), Gaithersburg, MD, USA). The NIST-certified concentration value for arsenic was 480 ± 100 μg/L, and the value measured in our laboratory was 474 ± 20 μg/L. The reliability of arsenic species separation was evaluated by the analytical recoveries of added arsenic species. The spiking of urine samples with 10 μg/L of iAs, MMA and DMA resulted in recoveries of 81%–92%, 88%–98% and 89%–103%, respectively. The concentrations of arsenic in the water samples were also determined using the above-mentioned method.

#### 2.2.2. Biochemical Analysis

The following common clinical biochemical variables were measured using a Hitachi autoanalyzer (Type 7170A; Hitachi; Ltd., Tokyo, Japan): triglyceride (TG), total cholesterol (TC), high-density lipoprotein cholesterol (HDL-C), low-density lipoprotein cholesterol (LDL-C), glucose (Glu), uric acid (UA), glycated hemoglobin (HbA1c), alanine aminotransferase (ALT), aspartate aminotransferase (AST), γ-glutamyl transpeptidase (γ-GT), and homocysteine (Hcy). Urinary creatinine was determined by the Jaffe reaction using a commercial kit (Nanjing Jiancheng Bioengineering Institute, Nanjing, China).

### 2.3. Statistical Analysis

The urinary concentration of total arsenic (tAs) was defined as the sum of the concentrations of iAs, MMA and DMA. The Mann-Whitney U test was performed to analyze the differences in biochemical variables between the subjects with and without arsenic exposure in the family. Fisher’s exact test was used to explore the differences in categorical variables between the subjects with and without arsenic exposure in the family. We used a *p* value of <0.05 to identify statistical significance.

### 2.4. Ethical Statements

All subjects gave their informed consent for inclusion before they participated in the study. The study was conducted in accordance with the Declaration of Helsinki, and the protocol was approved by the Ethics Committee of China Medical University (Identification code: CMU62073018).

## 3. Results

### 3.1. Chronic Arsenic Poisoning Diagnosed in the Household

**Figure 2:**
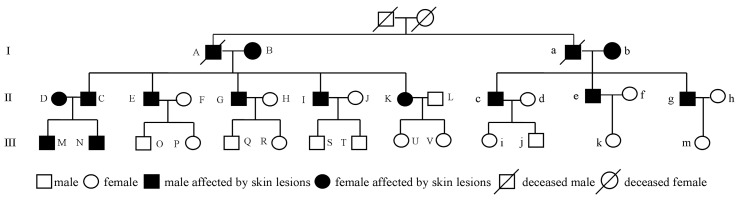
The pedigree tree of the family.

Figure 2 shows the pedigree tree of the family. Member “K” is the woman who was first identified. Members “A” and “a” are brothers who lived in the same household and died in 2009 and 1994, respectively. The cause of death of member “A” was cerebral hemorrhage and that of member “a” was skin cancer. Therefore, the household consisted of a total of 32 living members. Of them, four members (M, Q, R and j) worked outside of the village. Thus, detailed physical examinations of the remaining 28 family members were performed. Skin lesions similar to symptoms caused by chronic arsenic poisoning were detected in 12 members, including two in generation I, nine in generation II and one in generation III. According to the family members’ recollections, members “A”, “a” and “M” also presented with similar skin signs, while members “Q”, “R” and “j” lacked these skin manifestations.

**Figure 3:**
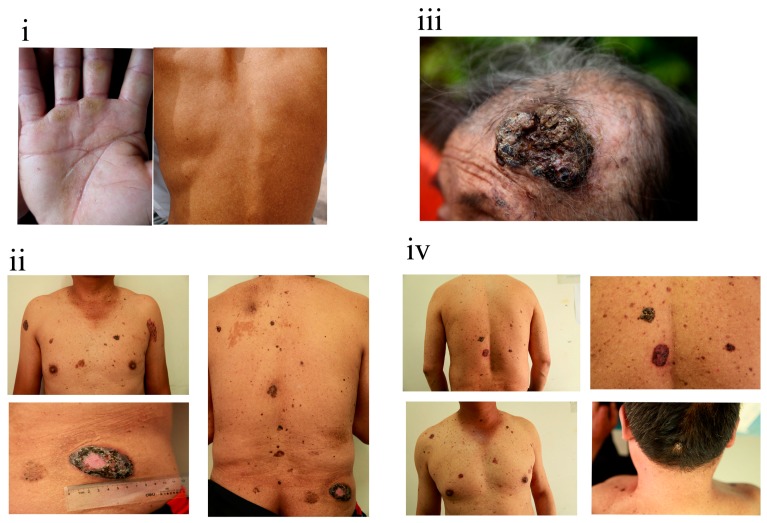
Clinical appearances of family members “N” (**i**); “G” (**ii**); “B” (**iii**) and “E” (**iv**).

The clinical severities of skin symptoms observed among the 12 individuals differed. As shown in Figure 3, pigmentation and de-pigmentation on the back and apparent hyperkeratosis on the palms were the major skin features in member “N” (Figure 3i). Apart from these skin lesions, family member “G” simultaneously presented with a number of bordered black-brown plaques on his chest and back (Figure 3ii). The size of the largest plaque, which was present on his hip, was approximately 6 cm. For family member “B”, a large excrescence occurred on the left side of her head (Figure 3iii). Similarly, gray-black excrescences were found on the back and head of member “E” (Figure 3iv). Based on the skin biopsy results, three individuals (b, C and E) were diagnosed with basal cell carcinoma, three (B, K and c) were diagnosed with Bowen’s disease, and two (G and I) were diagnosed with both basal cell carcinoma and Bowen’s disease. Only typical pathological features of hyperkeratosis were detected for family member “g”.

Considering the findings of the physical examinations and skin biopsies, it was possible to infer that the skin changes observed among these family members were associated with arsenic exposure. However, the family was currently obtaining drinking water from a tap water supply system that used surface water as a source. Arsenic concentration analysis showed that the arsenic levels in the drinking water were within the normal range (<10 μg/L). According to the urinary arsenic concentration test results, the median (25th, 75th) urinary values of iAs, MMA and DMA were 6.7 (4.3, 12.6) μg/L, 3.7 (3.0, 7.0) μg/L and 24.4 (18.8, 52.9) μg/L, respectively.

The median (25th, 75th) value of urinary tAs for these family members was 37.3 (25.4, 64.6) μg/L, which is within the normal range (<100 μg/L) suggested by the Agency for Toxic Substances and Disease Registry (ATSDR) for unexposed populations [21]. These findings provide additional evidence that the family members were not presently being exposed to arsenic.

**Figure 4:**
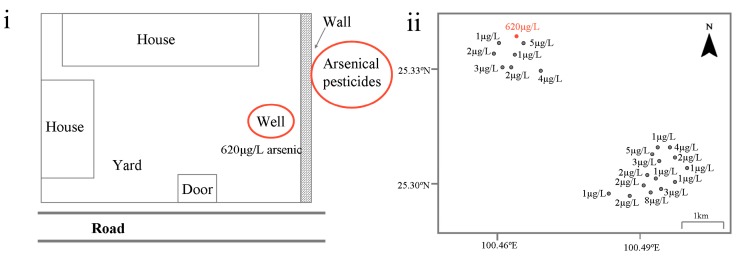
(**i**) The sketch of the family home (the red circles indicate the locations of arsenical pesticides and the well used to supply drinking water to the family from 1973 to 1989; the soil at the site where the arsenical pesticides were placed had been removed to pave the road in front of the door, which shown in bold); (**ii**) The geographical distribution and corresponding arsenic concentrations of the 21 groundwater samples collected near the family’s home (the red dot marked 620 µg/L represents the geographical location of the home).

Based on a careful review of the patients’ characteristics in the household, we found an interesting pattern: the 15 members affected by skin lesions were born or had moved into the household through marriage before 1989, and correspondingly, none of the 19 members who lived in the household after 1989 were affected (Table 1). After a thorough investigation and interview, the family members recalled that a large amount (approximately 2 t) of an arsenic-based pesticide known by the Chinese name “Shi Huang” (also known as “realgar”) had been previously placed in the vicinity of a well that supplied the household with drinking water from 1973 to 1989 (Figure 4i). The main components of the pesticide were As_4_S_4_ or As_2_S_2_. The pesticide was in powder form and packed in paper bags. No materials were used to cover these pesticides. In 1989, the large amounts of pesticides were completely removed because a neighbor wanted to build a small house at the site where it had been placed. The same year, the well stopped supplying drinking water to the family. Arsenic concentration testing of the well water was conducted immediately. As expected, the arsenic level was 620 μg/L, which was 62 times the maximum allowable level for arsenic in drinking water recommended by the World Health Organization (10 μg/L). However, it should be noted that the 620 µg/L arsenic in the well water might be a natural occurrence. Studies have found that the presence of naturally occurring arsenic in groundwater is determined by various geological and hydro-geochemical parameters, such as geology, climate, drainage, topography and soil type [22]. Thus, collecting additional groundwater samples near the family’s residence would be helpful for confirming whether the 620 µg/L arsenic in the well water was naturally occurring. As shown in Figure 4ii, a total of 21 groundwater samples near the residence were collected. The arsenic test results showed that the arsenic concentrations in these samples were normal, ranging from a minimum of 1 μg/L to a maximum of 8 μg/L (Figure 4ii). These findings provide further evidence that the excessive arsenic detected in the well water of the household was not a natural occurrence but that it was probably derived from the large amount of arsenical pesticides. Based on the above findings, it is possible to establish that the family members with skin lesions suffered from chronic arsenic poisoning and that the arsenical pesticides were probably responsible for the event.

According to above information, a total of 15 individuals in the family, including members “A” and “a”, had been exposed to high levels of arsenic. The exposure durations of these individuals varied significantly, from 16 years for 12 individuals to nine years for family member “D”, eight years for family member “M” and six years for family member “N” (Table 1). Moreover, family members “M” and “N” were exposed to arsenic as fetuses. Based on the diagnostic standard for endemic arsenicosis in China (WS/T211-2001), 10 individuals in the family were classified as advanced arsenicosis patients, and two were classified as mild arsenicosis patients (Table 1). At the time of this investigation, the family members had not used the arsenic-contaminated well water for nearly 24 years.

### 3.2. Persistent Health Influence

To fully understand the current physical conditions of the family members, some biochemical indexes commonly measured in clinical settings were tested. We found that the family members who had been previously exposed to arsenic had statistically significantly higher median AST and γ-GT levels than those who were unexposed (Table 2). The median level of urinary creatinine was higher for the subjects with arsenic exposure than those without (*p* < 0.05). A borderline significantly higher UA level was detected in the individuals who had been exposed to arsenic than in those who were unexposed (*p* = 0.074).

In addition, we compared the proportion of subjects with high ALT, AST, γ-GT and UA levels between the individuals who had been exposed and those who had not been exposed to arsenic. A high ALT (>40 U/L) level was only detected in family member “g”, who had been exposed to arsenic for 16 years. In contrast, 4 of the family members had abnormal AST (>40 U/L) and γ-GT (>50 U/L) levels, and they all had a history of arsenic exposure (Table 3). No significant difference in the proportion of individuals with a high UA level was found between the two groups (Table 3).

**Table 1:** General characteristics of the family members in the household (*n* = 34) ^£^.

Variables	Number	Birth Year ^#^	Skin Symptoms	Exposure Duration	Grade ^†^	Pathological Examination
Generation I	A *	1936	Yes	1973–1989 (16)	NA	NA
	B	1935 (NA)	Yes	1973–1989 (16) ^§^	Advanced	Bowen’s disease
	a *	1941	Yes	1973–1989 (16)	NA	NA
	b	1945 (1963)	Yes	1973–1989 (16)	Advanced	Basal cell carcinoma
Generation II	C	1956	Yes	1973–1989 (16)	Advanced	Basal cell carcinoma
	D	1958 (1980)	Yes	1980–1989 (9)	Mild	NA
	E	1963	Yes	1973–1989 (16)	Advanced	Basal cell carcinoma
	F	1965 (1990)	No	Non-exposed		
	G	1966	Yes	1973–1989 (16)	Advanced	Basal cell carcinoma/Bowen’s disease
	H	1969 (1991)	No	Non-exposed		
	I	1972	Yes	1973–1989 (16)	Advanced	Basal cell carcinoma/Bowen’s disease
	J	1976 (1996)	No	Non-exposed		
	K	1970	Yes	1973–1989 (16)	Advanced	Bowen’s disease
	L ^$^	NA	No	Non-exposed		
	c	1965	Yes	1973–1989 (16)	Advanced	Bowen’s disease
	d	1970 (1989)	No	Non-exposed		
	e	1970	Yes	1973–1989 (16)	Advanced	NA
	f	1974 (1997)	No	Non-exposed		
	g	1973	Yes	1973–1989 (16)	Advanced	Hyperkeratosis
	h	1976 (2000)	No	Non-exposed		
Generation III	M ^&^	1981	Yes	1981–1989 (8)	NA	NA
	N	1983	Yes	1983–1989 (6)	Mild	NA
	O	1993	No	Non-exposed		
	P	1997	No	Non-exposed		
	Q ^&^	NA	No	Non-exposed ^§^		
	R ^&^	NA	No	Non-exposed ^§^		
	S	1998	No	Non-exposed		
	T	2004	No	Non-exposed		
	U	1996	No	Non-exposed		
	V	2001	No	Non-exposed		
	i	1990	No	Non-exposed		
	j ^&^	NA	No	Non-exposed ^§^		
	k	1998	No	Non-exposed		
	m	2004	No	Non-exposed		

*Notes*: ^£^: information on founding parents of the family is not included in the table because they died before 1973; *: family member deceased; ^&^: family member works outside; ^#^: the number in the bracket is the year that the family member moved into the household through marriage; ^$^: this member is the husband of member K, and he has always lived in another village; §: the arsenic exposure status was confirmed based on the birth year of the next of kin.^†^ Based on the diagnosis standard for endemic arsenicosis in China (WS/T211-2001), the grade of arsenicosis was determined by the clinical severities of skin pigmentation and de-pigmentation on the trunk and hyperkeratosis on the palms of the hands and soles of the feet; NA: not available.

**Table 2:** Levels of biochemical indexes for family members exposed and not exposed to arsenic ^a^.

Biochemical Variables	Overall (*n* = 25)	Individuals Exposed to Arsenic (*n* = 12)	Individuals Unexposed to Arsenic (*n* = 13)	*p*-Value
ALT (U/L)	22.0 (16.0, 30.0)	27.5 (17.0, 30.8)	18.0 (13.0, 26.0)	0.185
AST (U/L)	34.0 (31.0, 38.0)	37.5 (33.0, 42.5)	31.0 (29.0, 34.0)	0.006
γ-GT (U/L)	14.0 (11.0, 34.0)	25.5 (13.3, 77.0)	12.0 (9.0, 19.0)	0.021
Creatinine (mg/dL)	102.0 (77.1, 163.1)	156.5 (108.7, 167.0)	77.4 (62.9, 127.7)	0.012
UA (μmol/L)	286.0 (201.0, 395.0)	353.5 (249.5, 396.5)	237.0 (183.0, 286.0)	0.074
Glu (mmol/L)	4.1 (3.8, 4.5)	4.1 (3.8, 4.7)	4.1 (3.6, 4.4)	0.518
HbA1c (%)	5.8 (5.7, 6.1)	6.1 (5.7, 6.1)	5.8 (5.5, 6.0)	0.245
TG (mmol/L)	1.5 (1.0, 1.6)	1.4 (1.0, 1.6)	1.5 (1.0, 1.8)	0.667
TC (mmol/L)	4.2 (3.9, 4.8)	4.2 (3.8, 5.6)	4.2 (4.0, 4.7)	0.580
HDL-C (mmol/L)	1.3 (1.2, 1.5)	1.4 (1.2, 1.5)	1.3 (1.2, 1.5)	0.599
LDL-C (mmol/L)	1.8 (1.6, 2.4)	1.7 (1.6, 2.9)	1.9 (1.6, 2.1)	0.902
Hcy (μmol/L)	14.0 (13.4, 16.8)	15.2 (13.7, 17.7)	13.7 (12.6, 16.2)	0.166

*Notes*: the data are expressed as the median (25th, 75th). *Abbreviations*: ALT, alanine aminotransferase; AST, aspartate aminotransferase; γ-GT, γ-glutamyl transpeptidase; UA, uric acid; Glu, glucose; HbA1c, glycated hemoglobin; TG, triglyceride; TC, total cholesterol; HDL-C, high-density lipoprotein cholesterol; LDL-C, low-density lipoprotein cholesterol; Hcy, homocysteine. ^a^ The Mann-Whitney U test was performed to analyze the differences in these biochemical indexes between the subjects with and without arsenic exposure in the family.

**Table 3:** Distribution of high AST, γ-GT and UA levels between family members who were exposed and unexposed to arsenic ^a^.

Variables	Individuals Exposed to Arsenic (*n* = 12)	Individuals Unexposed to Arsenic (*n* = 13)	*p*-Value
AST (U/L)			0.039
>40	4 (33.3)	0 (0.0)	
≤40	8 (66.7)	13 (100.0)	
γ-GT (U/L)			0.039
>50	4 (33.3)	0 (0.0)	
≤50	8 (66.7)	13 (100.0)	
UA (μmol/L)			0.593
HUA ^b^	2 (16.7)	1 (7.7)	
Normal	10 (83.3)	12 (92.3)	

*Notes*: data are expressed as number (%). *Abbreviations*: AST, aspartate aminotransferase; γ-GT, γ-glutamyl transpeptidase; UA, uric acid. ^a^ Fisher’s exact test was used to explore the differences in the variables between the two groups; ^b^ HUA is defined as a uric acid level of higher than 420 μmol/L in males and of higher than 360 μmol/L in females.

## 4. Discussion

It has been long recognized that arsenic-based pesticides are among the most common arsenical compounds used in agriculture. Most synthetic pesticides containing arsenic, such as lead arsenate, copper arsenate and calcium arsenate, were extensively produced after World War II [23]. In China, however, the practice of using arsenic in pesticides dates to the “Warring States Period,” when people used arsenical minerals to produce arsenic trioxide to kill rodents and to control silkworm diseases [24]. The arsenical pesticides placed near the well, which had previously supplied drinking water for the family, was a type of arsenical mineral known as realgar. Although the main components of realgar are As_4_S_4_ or As_2_S_2_, it naturally contains arsenic trioxide as an impurity [25]. Unlike As_4_S_4_ or As_2_S_2_, which have low solubilities, arsenic trioxide is easily dissolved in water. Experiments have shown that small amounts of soluble arsenic can be detected in realgar, even in that used for medication [26]. In addition, the realgar mentioned in our study had been crushed into powder by machines before being used as a pesticide. During this procedure, the main components, As_4_S_4_ or As_2_S_2_, were, at least partly, oxidized to arsenic trioxide due to the high temperatures used in this process. It should also be noted that no materials were used to cover the arsenical pesticides. Considering the above points, it is possible to infer that the soluble arsenic in the pesticides gradually permeated into the soil along with rainwater washout and then seeped into the nearby well. To some extent, collecting soil samples from around the site where the arsenic-based pesticides was placed was helpful for determining whether the excess arsenic in the well water was derived from these pesticides. Unfortunately, the soil at the particular site where the pesticides were placed was removed to pave the road in front of the family house and thus was not available (Figure 4i). However, no other high-arsenic wells were found near the family’s residence, providing evidence to conclude that the excessive arsenic in the well water was not a natural occurrence and correspondingly that the large amount of arsenical pesticides placed there is the most probable culprit. Furthermore, it is expected that the arsenic level in the well water during the period from 1973 to 1989 was higher than the current level (620 μg/L). Long-term consumption of well water contaminated with excessive arsenic consequently led to severe health outcomes, with approximate one-half of the family members exhibiting arsenic-related skin lesions.

Given the notorious adverse effects of arsenic exposure in humans, the United States Environmental Protection Agency (EPA) banned the use of many inorganic arsenic-based pesticides during the late 1980s and early 1990s [15]. In contrast, organic forms of arsenic (e.g., MMA and DMA) are considered less toxic and have been used as herbicides on agricultural lands, orchards and golf courses [15,27]. The use of organoarsenicals can also be a problem because a growing body of data indicates that there is a strong possibility that organic and inorganic forms of arsenic can be transformed via both biotic and abiotic processes [27,28,29]. Although some countries have issued documents to phase out organoarsenical pesticides from the market, large agricultural sites contaminated by years of organoarsenical pesticide application still exist. These agricultural lands might pose significant health risks in the present and in the future. Apart from its use in pesticides, arsenic is still allowed in the production of other materials, such as wood preservatives, glassware, and semi-conductors. The use of these synthetic arsenical compounds increases the possibility of arsenic exposure in humans. Therefore, the environmental effects of arsenical compounds should not be ignored; in fact, more attention should be paid to this issue, especially for people in direct contact with these arsenical products.

It is well recognized that long-term arsenic exposure can have multisystemic toxic effects, including skin, cardiovascular, neurological, respiratory, and developmental effects [30]. In particular, the skin is the most sensitive organ to arsenic exposure. Skin lesions (e.g., hyperkeratosis, pigmentation, and Mee’s lines on nails) and skin malignancies (e.g., Bowen’s disease and basal cell carcinomas) are usually the first visible signs of arsenic exposure and are considered to be the most prominent symptoms of chronic arsenic toxicity [31,32]. These skin manifestations can occur within months or several years after arsenic exposure and can persist for many years [33]. Therefore, a thorough physical examination for the presence of arsenic-related skin symptoms is invaluable to assess both recent and past arsenic exposure in field investigations. As shown in the current report, although the household had stopped drinking arsenic-contaminated well water by 1989, apparent arsenic-related skin manifestations were clearly observed in the arsenic-exposed individuals. More importantly, these skin characteristics provided important clues with which to trace the source of arsenic exposure in our study.

Several cross-sectional and case-control studies conducted in arsenic endemic areas of Taiwan, Bangladesh and India have suggested that the relationships between arsenic exposure and skin lesions and skin malignancies are in a dose-response manner [34,35,36,37,38,39]. A recent prospective study has also revealed that the dose-dependent association between arsenic exposure and skin lesions is present even at arsenic concentrations lower than 100 μg/L [2]. In our study, although the exact amount of arsenic ingested by each individual in the family was difficult to estimate, their arsenic exposure duration might reflect the cumulative arsenic dose. The 10 family members who were exposed to arsenic for 16 years had more severe skin manifestations, 8 of whom were diagnosed with skin cancer (Table 1). In contrast, the skin changes observed in family members “D” and “N”, who had arsenic exposure durations of 9 and 6 years, respectively, were relatively mild. The differences in skin changes observed among these family members might be related to the different arsenic exposure durations. Additionally, the findings might reflect the dose-response relationship between arsenic exposure and arsenic-related skin diseases.

Apart from the classical skin changes, liver injury is another common adverse effect induced by arsenic exposure [40,41]. A positive association between environmental arsenic exposure and abnormal liver function, as manifested by elevated ALT, AST and alkaline phosphatase (ALP) levels, has been reported [42]. Hepatomegaly, hepatoportal sclerosis, liver fibrosis and cirrhosis are also found much more frequently among populations with arsenic exposure compared to the general population [40,41,42,43]. A more recent meta-analysis has further proposed that arsenic exposure increases the risk of liver cancer mortality [43]. In this report, we also observed that subjects previously exposed to arsenic had significantly higher AST and γ-GT levels than those who were unexposed, although the family had not been exposed to arsenic for 24 years. These findings might indicate, at least to some extent, that the family members with arsenic exposure had liver dysfunction. However, it should be noted that the AST and γ-GT levels can be influenced by various factors, including age, body mass index, alcohol consumption and medication usage [44]. In this study, these influencing factors were not taken into consideration when analyzing the differences in the AST and γ-GT levels between the subjects exposed and not exposed to arsenic. Therefore, caution should be exercised in the interpretation of these findings. Additionally, as shown in Table 2, the urinary creatinine levels were significantly higher in the subjects exposed to arsenic than in the non-exposed subjects. Urinary creatinine is a reflection of hydration status, and it is widely used to adjust concentrations of urinary chemicals or their metabolites in spot samples [45]. Many factors, such as age, sex, body mass index and diet, have been shown to affect the urinary creatinine level [45]. These factors might have caused the differences in the urinary creatinine levels observed in our study.

There is no doubt that malignancies are the most serious health outcomes of arsenic exposure. These malignancies can occur either in the continuing presence of or after the cessation of exposure because of the long-term latency period for their development in humans [46]. A report of a Chilean cohort suggested that the lung and bladder cancer mortality rates in arsenic-exposed populations began to increase at approximately 10 years after the water arsenic levels increased, peaking at 10–20 years after the levels had decreased to the normal range [47]. Another well-known study that demonstrated the long latency period of arsenic-related malignancies is from Nakajo, Japan [48]. This study found that residents exposed to arsenic via well water for 5 years had an elevated risk of lung cancer at 34 years after the exposure ended. In contrast to the long average latency periods of internal cancers, as mentioned above, skin changes appear within a relatively short time period after exposure to arsenic, and accumulating evidence suggests that these skin changes can be considered precursors of arsenic-induced internal malignancies [49,50,51,52]. The associations between arsenical skin lesions and subsequent internal malignancies were first systematically explored by Cuzick *et al.* [50,51,52]. A cohort of 478 patients who had been given Fowler’s solution (containing 1% arsenic trioxide) for several weeks to 12 years was monitored for cancer mortality [50]. Excess bladder cancer mortality was reported in a 10-year follow-up study of arsenic-exposed patients with palm hyperkeratosis [50,51,52]. Another prospective study conducted in Taiwan also revealed that patients with skin cancer (Bowen’s disease and non-melanoma skin cancers) had significantly increased lung cancer (hazard ratio = 4.64, 95% confidence interval: 2.92–7.38) and urothelial carcinoma risks (hazard ratio = 2.02, 95% confidence interval: 1.23–3.30) after adjusting for confounding factors [49]. In the household examined in the current study, all of the members who had been exposed to arsenic from 1973 to 1989 presented with skin changes, either skin lesions or skin malignancies. Therefore, these individuals might be at a high risk of developing internal malignancy in the future. Based on this consideration, careful follow-up health examinations of the 13 living family members are crucial.

## 5. Conclusions

In this study, we have described a unique chronic arsenic poisoning event that occurred within a household in which approximately one-half of the family members were affected by skin changes. Although the family members had not been exposed to arsenic for 24 years, those who had been previously exposed to arsenic exhibited higher serum levels of AST and γ-GT than those who were unexposed. The family members with skin lesions and skin malignancies might be at a high risk of developing internal cancer in the future.

In modern society, the use of arsenic in industry and agriculture is very common. This report has elucidated the effects of arsenic-based compounds on the occurrence of high arsenic levels in drinking water. Environmental policymakers should formulate regulations to strengthen the management of these arsenic-related products.
